# Symmorphosis through Dietary Regulation: A Combinatorial Role for Proteolysis, Autophagy and Protein Synthesis in Normalising Muscle Metabolism and Function of Hypertrophic Mice after Acute Starvation

**DOI:** 10.1371/journal.pone.0120524

**Published:** 2015-03-25

**Authors:** Henry Collins-Hooper, Roberta Sartori, Natasa Giallourou, Antonios Matsakas, Robert Mitchell, Helen Mararenkova, Hannah Flasskamp, Raymond Macharia, Steve Ray, Jonathan R. Swann, Marco Sandri, Ketan Patel

**Affiliations:** 1 School of Biological Sciences, University of Reading, Whiteknights campus, Reading, United Kingdom; 2 Venetian Institute of Molecular Medicine, University of Padova, Padova, Italy; 3 Department of Food and Nutritional Sciences, School of Chemistry, Food and Pharmacy, University of Reading, Whiteknights campus, Reading, United Kingdom; 4 Centre for Cardiovascular and Metabolic Research, Hull York Medical School, Hull/York, United Kingdom; 5 Department of Cell and Molecular Biology, The Scripps Research Institute, La Jolla, California, United States of America; 6 Veterinary Basic Sciences, Royal Veterinary College, London, United Kingdom; 7 Natural Biosciences, University of Reading, Whiteknights campus, Reading, United Kingdom; University of Rome La Sapienza, ITALY

## Abstract

Animals are imbued with adaptive mechanisms spanning from the tissue/organ to the cellular scale which insure that processes of homeostasis are preserved in the landscape of size change. However we and others have postulated that the degree of adaptation is limited and that once outside the normal levels of size fluctuations, cells and tissues function in an aberant manner. In this study we examine the function of muscle in the myostatin null mouse which is an excellent model for hypertrophy beyond levels of normal growth and consequeces of acute starvation to restore mass. We show that muscle growth is sustained through protein synthesis driven by Serum/Glucocorticoid Kinase 1 (SGK1) rather than Akt1. Furthermore our metabonomic profiling of hypertrophic muscle shows that carbon from nutrient sources is being channelled for the production of biomass rather than ATP production. However the muscle displays elevated levels of autophagy and decreased levels of muscle tension. We demonstrate the myostatin null muscle is acutely sensitive to changes in diet and activates both the proteolytic and autophagy programmes and shutting down protein synthesis more extensively than is the case for wild-types. Poignantly we show that acute starvation which is detrimental to wild-type animals is beneficial in terms of metabolism and muscle function in the myostatin null mice by normalising tension production.

## Introduction

Starvation has plagued man from our prehistoric dawn leaving a mark on both the human psyche and genome undoubtedly due to its regularity and its devastating consequences. The most overwhelming loss of life due to starvation in modern times occurred about 50 years ago during the Great Leap Forward programme of social engineering in China with the death of 30 million people [1]. However the incidence of starvation is not confined to times of social instability or restricted to developing countries. There is a vast body of data that shows the prevalence of malnutrition and starvation in the general communities of Europe and USA especially amongst the venerable and aged populations [2].

The concept of ‘lethal weight loss’- the critical level of weight loss beyond which death becomes unavoidable has been explored by zoologists and physicians for almost two centuries. Some invertebrates can lose as much as 95% of their normal weight prior to death whereas in humans this figure is closer to 50% [3]. Importantly especially for Western societies, the lethal weight loss limit declines with age. However it is believed that lethal weight loss can be extended by increasing fat content and there are exceptional cases where obese individuals have maintained normal activity whilst undertaking a full-fasting regime for over 200 days [4]. Therefore in this scenario high levels of adiposity confer benefit. This seems to contradict the extensive volume of evidence supporting the belief that obesity is a negative health indicator for a number of conditions including diabetes, hypertension and stroke [5]. The resolution to the paradox was proposed by Neel in the Thrifty Gene Hypothesis (TGH) which proffers the idea that in the pre-modern times, when food shortages were extremely frequent, it was necessary to deposit fat to safeguard against starvation but that the same trait has become disadvantageous during modern times when food is plentiful in certain societies [6].

The degree of adaptation to stimuli is normally limited to a range very close to its normal size and composition. Such limitations are thought to be a universal feature of all normal tissues. This notion is incorporated in a process called symmorphosis which proposes that a biological design will be optimised such that structure is commensurate to normal function [7]. However tissues are not intended to respond to non-physiological parameters since this would incur costs in terms of development and maintenance. Symmorphosis is essentially an engineering principle, in which unnecessary spare capacity is eliminated and is a feature not only of biological processes but also everyday objects e.g. kitchen knives are manufactured to cut food not wood. The concept of symmorphosis is often difficult to test in a biological system as non-physiological adaptations frequently result in changes to multiple parameters [8]. However this hurdle has been overcome in a few genetic models that affect only one major tissue.

One classical adaptive process is undertaken by an organism during periods of starvation when the body fuels essential organs, especially the brain by drawing on the fat and muscle (with preference for the former over the latter). There is very good evidence both in rodents and humans that increasing levels of fat act to decrease the mobilisation of muscle protein as a source for fuel during starvation [9, 10]. Therefore the body reacts to starvation by protecting the muscle at the expense of fat, presumably because it confers survival advantage.

High organism protein content has always been depicted as a desirable status since it supports cellular function associated with immune system (e.g. white blood cells production of thymus and bone marrow), nutrient absorption, tissue repair and sexual processes (cellular, hormonal and psychological). Whilst this is substantiated by a wealth of data, it is imperative to recognise this concept only applies when muscle hypertrophies by relatively small levels above the norm, regulated by symmorphosis. This point is clearly exemplified through the numerous studies on the myostatin null mouse. Muscle enlargement takes place by both an increase in fibre number during embryonic development (hyperplasia) as well as pre and post natal fibre enlargement (hypertrophy) [11, 12]. Following germ-line deletion of myostatin, the mass of individual skeletal muscles can increase by 200% and accompanied by a near total loss in body fat. However a number of studies have shown that the hypertrophic muscle of myostatin null mice has impaired force production characteristics and that mutant animals have lower exercise capacity [13–16]. The decrease in specific force (tension divided by mass) is indicative of aberrant muscle physiology a feature that is also displayed during the ageing process and in myopathies [17].

In this study we examined the consequence of acute starvation in the myostatin null mouse. We show that the untreated (i.e. under ad libitum rodent diet) hypertrophic muscle had a metabolic profile indicative of glycolytic based energy production programme, conducive to tissue growth and elevated baseline levels of a novel signalling pathway that supports protein synthesis revolving around Serum/Glucocorticoid Kinase 1 (SGK1) and that the levels of Akt, the canonical regulator of protein synthesis are actually lower in the myostatin null mouse. Surprisingly we also found evidence of increased levels of autophagy in resting muscle of the myostatin null mouse. We show that starvation leads to similar levels of body mass loss in both the wild type and mutant genotypes but that weight loss originates from differing tissues. In the myostatin null we show elevated loss of muscle compared to wild-types and that this occurs through a unique combination of decreased protein synthesis as well as through autophagy and proteosome mediated peptide breakdown. Significantly we show that these changes result in the normalisation of myostatin muscle metabolism and function.

## Materials and Methods

### Ethical approval

All research conducted on animals was performed under license from the United Kingdom Home Office in agreement with the Animals (Scientific Procedures) Act 1986.

### Animal maintenance

*Mstn* ^*−/−*^ mice were a kind gift from Se-Jin Lee (John’s Hopkins USA). Healthy male C57Bl/6 and *Mstn* ^*−/−*^ mice were bred and maintained according to the NIH Guide for Care and Use of Laboratory animals, approved by the University of Reading Animals were housed in the Biological Resource Unit under standard environmental conditions (20–22°C, 12h–12h light–dark cycle) and provided food and water *ad libitum*. All mice were of 4 months of age at the commencement of the starvation diet.

### Starvation protocol

Mice were moved from normal cages to ones containing a water source as well as copious inedible bedding material to mitigate any effects of hypothermia. Animals were monitored every 6 hours and maintained under starvation conditions for a maximum of 24h.

### Puromycin administration

Mice were injected exactly 30 minutes before tissue collection with 0.04 μmol/g body mass puromycin into the peritoneal cavity and then returned to their cages.

### Histological analysis and Immunohistochemistry

Mice were euthanized according to Schedule 1 of the Animals Scientific Procedures Act (UK). All tissue was kept moist with PBS during the muscle dissection process which following resection was immediately frozen in liquid nitrogen-cooled isopentane. Muscles were thereafter mounted in Tissue Tech freezing medium (Jung) cooled by dry ice/ethanol. Immunocytochemistry was performed on 10 μm cryosections which were dried for 30 minutes before the application of block wash buffer (PBS with 5% foetal calf serum (v/v), 0.05% Triton X-100). All antibodies were diluted in wash buffer 30 minutes before use. Myosin Heavy Chain (MHC) type I, IIA and IIB isoforms were identified by using A4.840 IgM (1:1 dilution), A4.74 IgG (1:4 dilution) and BF-F3 IgM (1:1 dilution) supernatant monoclonal primary antibodies (Developmental Studies Hybridoma Bank). Antibodies against SGK, pSGK1 (Cell Signalling), puromycin (Millipore) and p62 were used at concentrations of 1:500, 1:500, 1:5000 and 1:1000 for respectively. Primary antibodies were visualized using Alexa Fluor 488 goat anti-mouse IgG (Molecular Probes A11029, 1:200) and Alexa Fluor 633 goat anti-mouse IgM (Molecular Probes A21046, 1:200) secondary antibodies.

### SDH staining

Muscle sections were briefly incubated for 30 minutes at 37°C in a sodium phosphate buffer containing 75mM sodium succinate (Sigma), 1.1mM nitroblue tetrazolium (Sigma) and 1.03mM phenazine methosulphate (Sigma). Samples were then fixed in 10% formol-calcium and cleared in xylene prior to mounting with DPX mounting medium (Fisher). Photographic quantification of the samples was performed on a Zeiss Axioskop2 microscope mounted with an Axiocam HRc camera. Axiovision Rel. 4.8 software was used to capture the images.

### Quantitative RT PCR

Total RNA from the TA was prepared using Promega SV Total RNA Isolation kit from which cDNA was synthesised using Invitrogen SuperScript III Reverse Transcriptase. Primer Express 3.0 (Applied Biosystems) was used to design PCR primers (Table 1 in S1 Dataset) which were used together with the Qiagen QuantiTect SYBR Green PCR Kit for quantitative RT-PCR (qRT PCR). All data were normalized to GAPDH expression.

### Western blotting

Muscle proteins were solubilised in lysis buffer (40 mmol Tris pH 7.5, 1 mmol EDTA, 5 mmol EGTA, 0.5% Triton X100 and protease inhibitors (Calbiochem 539131)). For qualitative analysis, 20 μg of protein was resolved on a 4–12% SDS PAGE before transferring to a PVDF membrane. Quantification of total puromycin incorporation into polypeptides was performed using a slot blotter by immobilising 6 μg of total solubilised muscle protein.

Membranes were probed for puromycin (mouse anti-puromycin ab 1:1000, Millipore MABE343), SGK1 pSGK1(rabbit anti SGK ab 1:2500, rabbit anti pSGK ab 1:2500 Cell Signalling 3272) followed by incubation with Horseradish peroxidase-conjugated goat anti-mouse or goat anti-rabbit secondary antibodies (R&D Systems). Visualization of the proteins were carried out by using enhanced chemiluminescence (ECL, GE Healthcare RPN3000OL1) and bands quantified by laser densitometry using Gel-Pro Analyzer 6.0 software.

### Muscle tension measurements

Dissection of the hind limb was carried out under oxygenated Krebs solution (95% O_2_ and 5% CO_2_). Under circulating oxygenated Krebs solution one end of a silk suture was attached to the distal tendon of the EDL and the other to a Grass Telefactor force transducer (FT03). The proximal tendon remained attached to the tibial bone. The leg was pinned to a Sylgard-coated experimental chamber. Two silver electrodes were positioned longitudinally on either side of the EDL. A constant voltage stimulator (S48, Grass Telefactor) was used to directly stimulate the EDL which was stretched to attain the optimal muscle length to produced maximum twitch tension (*P*_t_). Tetanic contractions were provoked by stimulus trains of 500 ms duration at, 10, 20, 50, 100 and 200 Hz. The maximum tetanic tension (*P*_o_) was determined from the plateau of the frequency-tension curve.

### ^1^H NMR spectroscopy-based metabonomic analysis

Polar metabolites were extracted from gastrocnemius muscle using the protocol previously described by Beckonert *et al*. [18]. Briefly, 40–50 mg of muscle tissue was snap frozen in liquid nitrogen and finely ground before further homogenization in 300μL of chloroform:methanol (2:1) using a tissue lyzer. The homogenate was combined with 300μL of water, vortexed and spun (13,000 g for 10 minutes) to separate the aqueous (upper) and organic (lower) phases. A vacuum concentrator (SpeedVac) was used to remove the water from the aqueous phase before reconstitution in 550 μL of phosphate buffer (pH 7.4) in 100% D_2_O containing 1 mM of the internal standard, 3-(trimethylsilyl)-[2,2,3,3,-^2^H_4_]-propionic acid (TSP). For each sample, a standard one-dimensional NMR spectrum was acquired with water peak suppression using a standard pulse sequence (recycle delay (RD)-90°-*t*_1_–90°-*t*_m_-90°-acquire free induction decay (FID)). RD was set as 2 s, the 90° pulse length was 16.98 μs, and the mixing time (*t*_m_) was 0.01 ss. For each spectrum, 8 dummy scans were followed by 128 scans with an acquisition time per scan of 3.8 s and collected in 64K data points with a spectral width of 12.001 ppm. ^1^H NMR spectra were manually corrected for phase and baseline distortions and referenced to the TSP singlet at δ 0.0. Spectra were digitized using an in-house MATLAB (version R2009b, The Mathworks, Inc.; Natwick, MA) script. To minimize baseline distortions arising from imperfect water saturation the region containing the water resonance was excised from the spectra. Principal components analysis (PCA) was performed with Pareto scaling in MATLAB using scripts provided by Korrigan Sciences Ltd, UK.

### Cross-sectional area measurements

Immunofluorescent stained muscle sections were photographed under a 10X lens on a Zeiss Axioimager A1 microscope using a Zeiss Axiocam camera. Three different photographs were taken at random on three different sections per muscle per animal. The interactive measurements tool in the Axiovision Rel 4.8 software was then used to process the images to measure cross-sectional area. All muscle fibres per fibre type were measured per field of view of the photographs taken (approximately 200 in total).

### Statistical analysis

Data are presented as mean ± SD. Significant differences on body weight between groups was determined using multivariate analysis of variance (MANOVA) followed by Bonferroni’s post-hoc test with repeated measurements with time. Wherever applicable, comparisons between two groups were performed by Student’s t-test for independent variables. Differences among groups were analysed by one-way ANOVA followed by Bonferroni’s multiple comparison tests. Differences were considered statistically significant at p<0.05.

## Results

### Starvation reduces body mass and skeletal muscle mass

**Fig 1:**
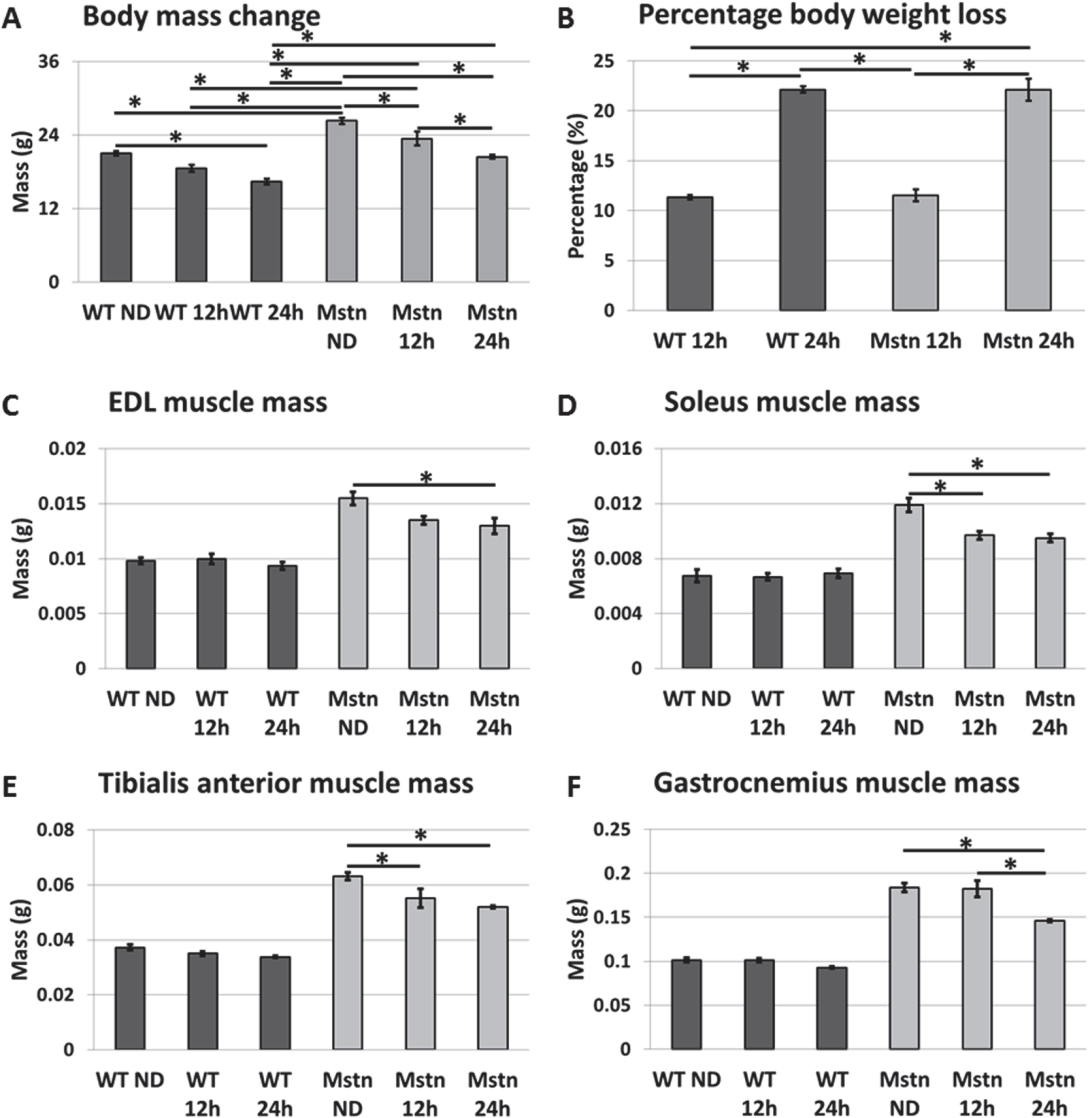
Changes in body mass in wild-type (WT) and *Mstn* ^*−/−*^ (Mstn) at 12 and 24 hours after complete food withdrawal (acute starvation). (A) Wild-type mice showed a significant decrease after 24h starvation. In contrast *Mstn* ^*−/−*^ lost mass in the first and second 12h of starvation. (B) Changes in body weight expressed as a % of normal body weight. Weight changes of (C) EDL, (D) soleus, (E) TA and (F) Gastrocnemius at 12 and 24h after acute starvation. Wild-type animals showed no significant muscle mass reduction in contrast to all muscles examined from *Mstn* ^*−/−*^. (Two-way ANOVA; *p<0.05)

To explore the effect of acute dietary withdrawal on hypertrophic muscled mice, all food was withheld for a maximum period of 24 hours. As expected at the commencement of the experiment, the *Mstn* ^*−/−*^ animals were heavier than age and sex matched wild-type mice. Both cohorts lost the same relative mass at 12 and 24 hours of food-deprivation (Fig 1A–1B and Table 2a in S1 Dataset). We then examined the contribution of skeletal muscle to body mass loss. To develop a comprehensive picture for the effect of starvation on muscle mass the weights of the Soleus, Tibialis Anterior (TA), Extensor Digitoum Longus (EDL) and Gastrocnemius were taken which revealed a clear difference between the genotypes. Wild-type animals displayed a small decrease in mass after 12h ranging from 1–6% across the muscles examined that failed to reach significance (Fig 1C–1F and Table 2b in S1 Dataset). In contrast the *Mstn* ^*−/−*^ had lost up to 18% muscle mass. During the second 12h period, wild-type muscle mass decreased by a maximum of 9%. However, even at this stage the decrease in wild-type muscle weights did not reach significance. In contrast the degree of muscle mass loss in the *Mstn* ^*−/−*^ cohort was significantly higher for all muscles examined. Therefore the *Mstn* ^*−/−*^ mice lose muscle not only earlier but also for prolonged periods.

### Starvation induced muscle fibre hypotrophy

Having shown genotypes a differential response to starvation in terms of muscle loss we next investigated the contribution of specific fibre types in this process. It is worth bearing in mind that *Mstn* ^*−/−*^ deletion causes a shift from slow myosin to fast isoforms. To that end we profiled fibre number and fibre type cross sectional area for the EDL, Soleus and TA. There was no change in total muscle fibre number or change in fibre type proportions after a 24-hour period of starvation in any of the muscles form either genotype in any muscles examined (data not shown).

**Fig 2:**
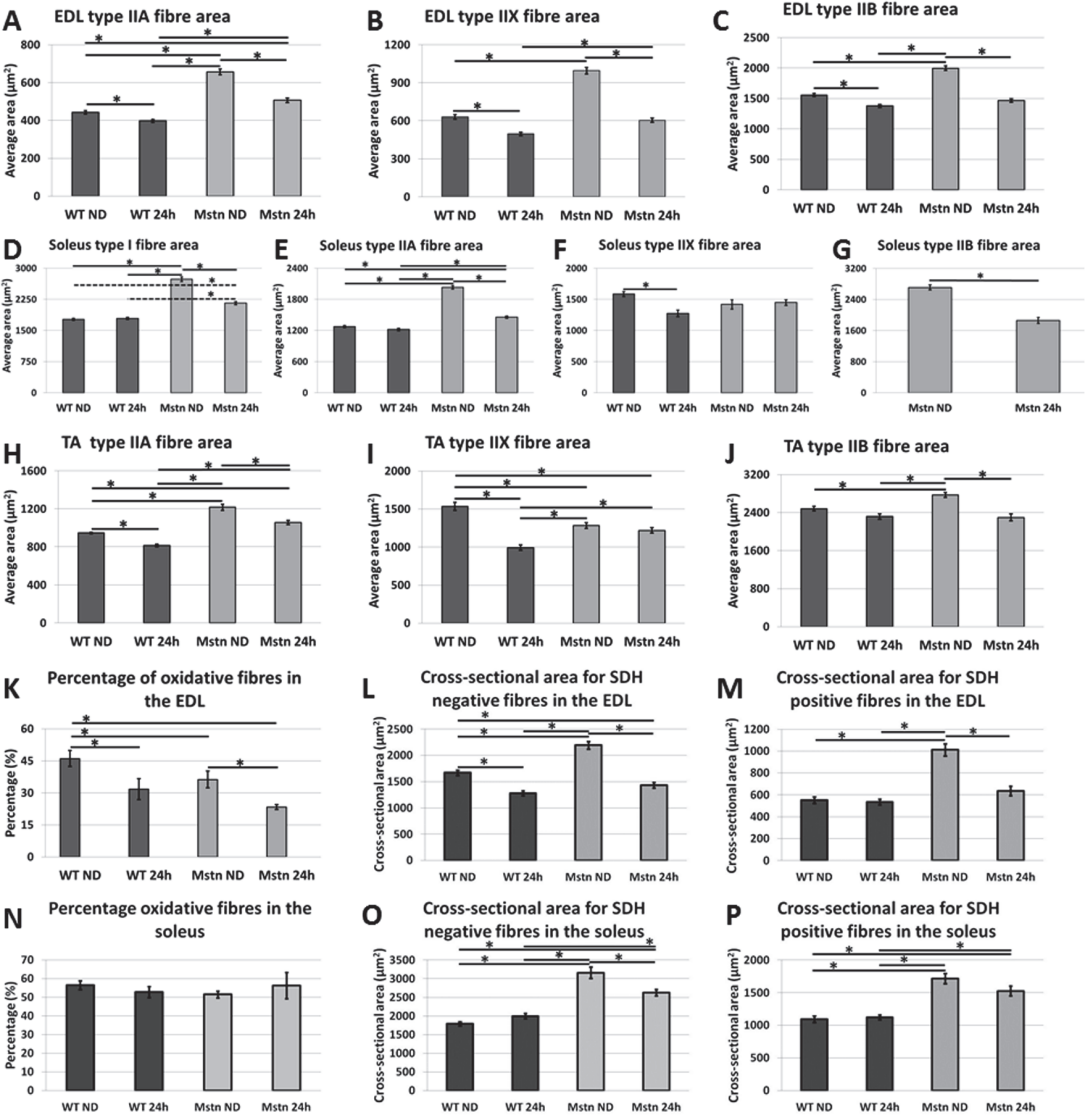
Fibre type profiling after acute starvation. MHC fibre cross sectional area of Wild-type and *Mstn* ^*−/−*^ EDL (A-C), Soleus (D-G) and Tibialis Anterior (TA) (H-J) before and 24h after starvation of wild-type and *Mstn* ^*−/−*^ mice. (K) Percentage of SDH positive (oxidative) and SDH negative fibres from the EDL of wild-type and *Mstn* ^*−/−*^ mice. (L) Changes in fibre diameter of SDH negative fibres of the EDL in wild-type and *Mstn* ^*−/−*^ mice before and after starvation. (M) Changes in fibre diameter of SDH positive fibres of the EDL in wild-type and Mstn mice before and after starvation. (N) Percentage of SDH positive (oxidative) and SDH negative fibres from the Soleus of wild-type and *Mstn* ^*−/−*^ mice. (O) Changes in fibre diameter of SDH negative fibres of the soleus in wild-type and *Mstn* ^*−/−*^ mice before and after starvation. (P) Changes in fibre diameter of SDH positive fibres of the soleus in wild-type and Mstn mice before and after starvation. (Two-way ANOVA; *p<0.05).

Examination of EDL fibre CSA revealed hypertrophy in all MHC types (IIA, IIX and IIB) in normal diet fed *Mstn* ^*−/−*^. Starvation for a period of 24h resulted in a significant decrease in all three fibre types irrespective of genotype (Fig 2A–2C and Table 3a in S1 Dataset). However there was a smaller decrease in the CSA for each specific fibre-type in the wild-types (-10% IIA, -21% IIX, -11%IIB) compared to *Mstn* ^*−/−*^ (-22% IIA, -37% IIX, -27%IIB). The most prominent result from the analysis of the EDL was the change in the size of the IIB fibres in the *Mstn* ^*−/−*^ following 24h starvation. These fibres decreased in size to such an extent that they were no longer significantly different in size compared to those from starved wild-type animals.

The soleus displayed an interesting feature not seen in the EDL. Here it is only those fibres that have undergone hypertrophy as a consequence of myostatin deletion that decreased in size (-21% I, -27% IIA, -31% IIB see Fig 2D–2G and Table 3b in S1 Dataset). The most prominent muscle fibre types in the wild-type (I and IIA) did not show any significant decrease in size. Of note was the finding that the type IIX fibres (which are of the same size in both genotypes) underwent a significant starvation-mediated hypotrophy (-19%) only in the wild-type mice.

Examination of the TA (the muscle with the least difference in the mass decrease after starvation) showed a significant decrease in CSA in both the IIA and IIX fibres in both genotypes and the IIB of the *Mstn* ^*−/−*^ (Fig 2H–2J and Table 3c in S1 Dataset). The major difference in terms of fibre type decrease to explain the mass loss was the 17% change in numerous type IIB fibres in the *Mstn* ^*−/−*^ compared to the 8% loss in the wild-types.

Next we examined the muscle for changes in metabolic properties induced by starvation by quantifying the number of fibres expressing SDH. As expected the fed wild-type EDL contained a significantly greater proportion of SDH expressing fibres than the fed *Mstn* ^*−/−*^ (46% vs. 36% respectively, Fig 2K and Table 3d in S1 Dataset). After 24h of starvation there was significant decrease in the number of SDH positive fibres in both groups. However the decrease was greater in the wild-types compared to the *Mstn* ^*−/−*^ (-32% v 22% respectively) such that there was no longer a statistical difference in the number of SDH positive fibres after starvation. There was no change in the profile of oxidative versus non-oxidative fibres in the soleus (Fig 2N). We next examined if there was a relationship between metabolic activity and atrophy in the two genotypes. Here we found that in the EDL wild-type, SDH negative fibres decreased in size after starvation while there was no change in SDH positive fibres. The EDL muscle of the *Mstn* ^*−/−*^ however showed a significant decrease in both populations but with a greater reduction in CSA of the SDH negative fibres (Fig 2L–2M). In the soleus, there was no change in the CSA of SDH^+^ or SDH^−^ fibres in wild-type after starvation, whereas we found a decrease only in the SDH^−^ fibres of *Mstn* ^*−/−*^ (Fig 2O and 2P). This suggests that glycolytic (i.e. non-oxidative) fibres were more susceptible in losing CSA after acute starvation.

### Molecular profiling of key determinants of proteolysis and autophagy after starvation

**Fig 3:**
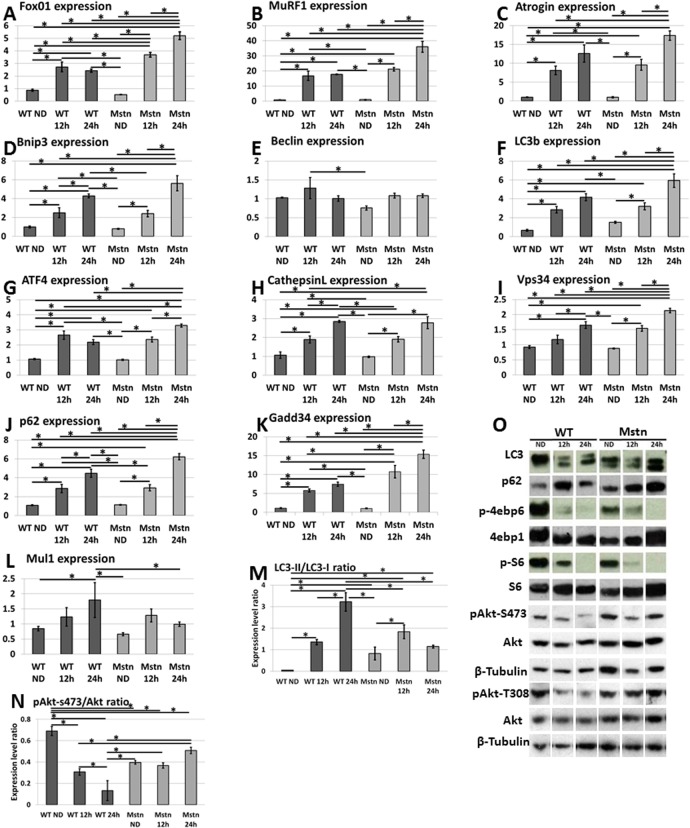
Molecular and protein analysis of the Tibialis Anterior (TA) for catabolic and anabolic markers in response to acute starvation. (A) qPCR analysis of changes in the expression of master regulator of catabolism, *Foxo1*. (B-C) E3 class of ubiquitin ligases, *MuRF1* and *atrogin*. Regulators of autophagy (D) *Bnip3*, (E) *Beclin*, ((F) *LC3b*, (G) *ATF4*, (H) *CathepsinL* (I) *Vsp34* (J) *p62*, (K) *Gadd34*. (L) mRNA expression of *Mul1* a mitophagy regulator. All qPCR levels normalised to levels of G6PDH. (M) Quantification of LC3-II in relation to LC3 at protein level. (N) Quantification of pAkt at residue S473 in relation total Akt at protein level. (O) Western blot analysis of regulators of autophagy (LC3 and p62), and protein synthesis (Akt-total and phosphorylated versions at S473 and T308, 4ebp1 and S6) with tubulin serving as loading control. (Two-way ANOVA; *p<0.05).

Muscle mass is regulated by mechanisms that control protein breakdown and protein synthesis. To gain an insight into the mechanisms that control muscle loss in our hypertrophic model we first examined the expression of *FoxO1* a master regulator that activates catabolism through proteolysis and autophagy. We found that starvation induced *FoxO1* in both genotypes. However there was a profound difference in the nature of the increase both in terms of degree and time (Fig 3A). Firstly, *FoxO1* was more robustly increased in the *Mstn* ^*−/−*^ after 12h compared to wild-type. Secondly whereas there was no subsequent increase in the level of *FoxO1* in the second 12h of starvation in wild-type mice, there was a significant increase in the *Mstn* ^*−/−*^ mice.

We next investigated the expression of key regulators of proteolysis by quantifying the expression of *MuRF1* and *Atrogin*, which encode for E3 ubiquitin ligases [19]. Their expression was significantly increased in both genotypes after the first 12h of starvation (Fig 3B–3C). However in the next 12h, the levels of both genes remained stable in the wild-type but increased significantly in the *Mstn* ^*−/−*^. Therefore there is a more robust and prolonged induction of genes that regulate protein breakdown through the proteosome in the *Mstn* ^*−/−*^ animals.

Next we examined the expression of genes that control protein breakdown through autophagy. To that end we quantified the levels of *Bnip3* (inducer of autophagy), Beclin (which promotes the development of the autophagosome, the double membrane vacuole that develops around structures targeted for breakdown), *LC3b* (a protein that controls the development of the membrane component of the autophagosome through its ability to undergo lipidation with Phosphatidylethanolamine), *ATF4* (a cysteine protease responsible for cleaving *LC3* at its C-terminal arginine residue facilitating the exposed C-terminal glycine to be conjugated to Phosphatidylethanolamine), *cathepsin L* (a key lyosomal cysteine endopepdidase required for the degradation of autophagosomal content), *Vsp34* (a class III phosphoinositide-3-kinase (PI3K), assembles into multi-protein complexes that include Beclin1), *p62* (a receptor for cargo destined to be degraded by autophagy) *Gadd34* (a protein phosphatase 1 regulatory subunit that induces autophagy by supressing the mTOR pathway [20]. In wild-type animals, starvation for 12h resulted in an induction of 6 of the 8 autophagy genes (all except *Beclin* and *Vsp34*) (Fig 3D–3K). During the same period all genes except *Beclin* were induced in the muscle from *Mstn* ^*−/−*^ animals. During the second 12 hours of starvation there was an increase in the expression of only 4 of the 8 genes (*p62*, *Bnip*, *Cathepsin L* and *Vsp34*) in the wild-type muscle compared to increases in all but two of eight in the muscle from *Mstn* ^*−/−*^ animals. In terms of quantity, at 12h there was greater expression of only *Gadd34* in the *Mstn* ^*−/−*^ muscle compared to wild-type muscle. However by 24h the situation had changed markedly with 5 genes being more highly expressed in *Mstn* ^*−/−*^ muscle compared to wild-type (*Gadd34*, *Lc3b*, *p62*, *ATF4* and *Vps34*).Therefore in a manner similar to the proteolysis regulators, the expression of autophagy mediators is both enhanced and sustained longer in muscle from *Mstn* ^*−/−*^ animals compared to wild-type after starvation.

We extended these studies by investigating the expression of key markers of autophagy at the protein level since post-translational modification plays a major role in this catabolic process. One of the key indicators of autophagy is the lipidation of LC3 which is generated during the formation of the autophagosome and is a read-out of autophagic activity. Although LC3 was present in the non-starved muscle of both genotypes, there was a major difference in the abundance of the lipidated form; it was scarce in wild-type muscle but present in significant amounts in *Mstn* ^*−/−*^ muscle (Fig 3M and 3O). These results show that basal levels of autophagy are higher in the *Mstn* ^*−/−*^ compared to wild-type muscle. A similar pattern was detected for p62 (quantification shown in Table 4a in S1 Dataset). Starvation in the first 12 hours induces similar levels of LC3-II in both genotypes. LC3-II levels continued to increase relative to LC3-I in wild-types during the second 12h period of starvation. Interestingly the ratio does not show a similar increase in the second 24h in the *Mstn* ^*−/−*^ even though the LC3-II expression is greatly increased. This could be due to increased expression of LC3 as shown by RT-PCR (Fig 3F and Table 4b in S1 Dataset).

We also examined the expression of *Mul1*, a gene that encodes a master regulator of mitophagy [21]. Here we found an increase in gene expression only wild-type muscle after a period of 24h and not a significant change in that of *Mstn* ^*−/−*^ tissue at any time point (Fig 3L and Table 4c in S1 Dataset).

### The effect of starvation on key regulators of protein synthesis

Akt a serine/threonine-specific protein kinase (also known a Protein kinase B) is a nodal molecule in muscle homeostasis as regulates both anabolic and catabolic processes. Akt levels normally suppress both autophagy and ubiquitin mediated protein breakdown by supressing *FoxO1* (Table 5a in S1 Dataset). Our molecular profiling data showed that *FoxO1* was robustly induced in the muscle of *Mstn* ^*−/−*^ mice with the concomitant increase in key indices of autophagy and ubiquitin proteolysis. We profiled the abundance of the active form of Akt (phosphorylated version at residues S473 or T308) and found that in the wild-type muscle, levels of pAkt fell in significant incremental stages during the starvation protocol. Surprisingly, there was no significant change in the levels of pAkt during any stage of starvation in the *Mstn* ^*−/−*^ animals (Fig 3N and 3O and Table 4b in S1 Dataset).

We next examined the activity of key proteins, ribosomal protein S6 (S6) and eukaryotic translation-initiation factor 4E-binding protein (4ebp1) that regulate protein synthesis and are believed to be targets of Akt activity. We found that the activity of both proteins was reduced in both genotypes in incremental stages during the starvation procedure (Fig 3O, Table 5b-d in S1 Dataset and Fig A-C in S1 Fig).

These results show that there is an uncoupling of Akt/protein synthesis pathway in the muscle of *Mstn* ^*−/−*^ mice.

### Muscle protein synthesis and starvation

Our data showed seemingly contradicting results in terms of regulators of protein synthesis (sustained activated Akt levels) but reduced levels of its target protein S6 and 4ebp1) in the TA from *Mstn* ^*−/−*^ animals. We therefore investigated the degree of protein synthesis by using SUnSET [22]. This is a nonradioactive method to follow polypeptide production based on the ability of Puromycin, an aminonucleoside antibiotic to become incorporated into nascent proteins. To monitor protein synthesis in muscle we injected puromycin into the peritoneal cavity exactly 30 minutes before killing the mice for muscle extraction.

**Fig 4:**
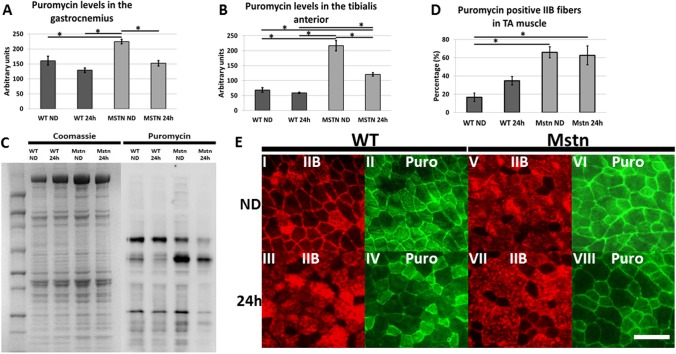
Quantitative and qualitative analysis of protein synthesis in wild-type and *Mstn* ^*−/−*^ Tibialis anterior and gastrocnemius muscle using SUnSET. (A-B) Slot-blot western analysis for quantification of protein synthesis in the gastrocnemius and TA muscle. (C) Western blot showing total protein distribution and puromycin labelled polypeptides from wild-type and *Mstn* ^*−/−*^ TA muscle. Note that some bands remain of similar intensity whereas others decrease. (D) Altered profile of high levels of protein synthesis in IIB fibres of *Mstn* ^*−/−*^ TA muscle. (E) Immunochistochemistry for the expression of MHC IIB and high levels of protein synthesis determined by puromycin incorporation. (Scale bar = 100μm). (Two-way ANOVA; *p<0.05)

We profiled the distribution of the pulsed puromycin in muscle to gauge the relationship between fibre-type and protein synthesis and found a significant difference in the activity between the genotypes. In wild-type TA muscle less than 20% of the type IIB fibres showed high levels of protein synthesis in contrast to the 70% in *Mstn* ^*−/−*^ mice (Fig 4D). This distribution did not change significantly after 24h starvation in either genotype (Fig 4D and 4E). Western blot profiling of polypeptides showed incorporation of puromycin into a numerous proteins that had been synthesised during the 30 minutes after injection (Fig 4C). Most but not all proteins from both genotypes showed a decrease in abundance as a consequence of starvation. We next quantified the levels of puromycin in a given amount of protein to determine relative levels of protein synthesis in muscle using slot blotting followed by anti-puromycin antibody binding and scanning densitometry. Our analysis of both the TA and gastrocnemius showed that protein synthesis decreased in wild-type muscle following starvation but not to significant levels (-15% and -19% respectively). The levels of puromycin incorporation was significantly higher in both muscles from fed *Mstn* ^*−/−*^ animals but that the level fell significantly after starvation (-44% and -32% respectively Fig 4A and 4B and Table 6a in S1 Dataset). These results show that decreased protein synthesis plays a greater role in the muscle catabolic process induced by starvation in the *Mstn* ^*−/−*^ animals compared to wild-types.

### Metabonomic analysis of gastrocnemius muscle

**Fig 5:**
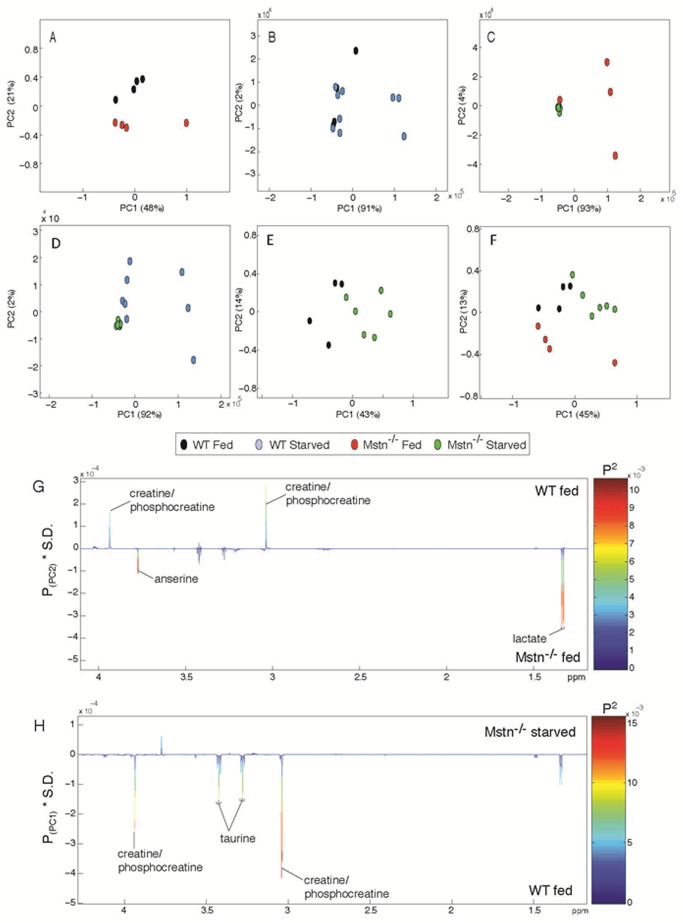
Pair-wise comparisons of the metabolic profiles obtained from gastrocnemius muscle from wild-type and *Mstn* ^−/−^ mice under fed and starved states. PCA scores plots (PC1 vs PC2) comparing (A) wild-type fed vs *Mstn* ^−/−^ fed; (B) wild-type fed vs wild-type starved; (C) *Mstn* ^−/−^ fed vs *Mstn* ^−/−^ starved; (D) wild-type starved vs *Mstn* ^−/−^ starved; (E) wild-type fed vs *Mstn* ^−/−^ starved; (F) wild-type fed vs *Mstn* ^−/−^ fed vs *Mstn* ^−/−^ starved (% variance explained in the parenthesis). Colour loadings plots shown for (G) PC2 of the model comparing wild-type fed vs *Mstn* ^−/−^ fed and (H) PC1 of the model comparing wild-type fed vs *Mstn* ^−/−^ starved. Product of PC loadings with standard deviation of the entire data set coloured by the square of the PC loading.

Metabolic profiles were acquired from the hydrophilic extracts of the gastrocnemius muscle by ^1^H nuclear magnetic resonance (NMR) spectroscopy. Principal components analysis (PCA) was applied to these profiles to identify metabolic variation in the muscle tissue associated with genotype and diet. The scores plot from the pair-wise PCA model comparing wild-type and *Mstn* ^−/−^ fed muscle revealed basal metabolic differences between the two genotypes (Fig 5A). This variation was observed in the second principal component and is explained by greater lactate and anserine in the hypertrophic *Mstn* ^−/−^ muscle compared to that of wild-type animals and lower amounts of creatine/phosphocreatine (Fig 5G). Starvation induced a varied metabolic response in the muscles of wild-type mice (Fig 5B). Minimal metabolic perturbation was observed in the majority of these animals but in a small subset (*n* = 3) starvation increased the amount of lactate, creatine/phosphocreatine and taurine in the muscle (Fig A in S2 Fig). In stark contrast, the metabolic response of *Mstn* ^−/−^ muscle to starvation was highly consistent across all group animals characterized by reductions in muscular lactate, creatine/phosphocreatine and taurine compared to the *Mstn* ^−/−^ fed mice (Fig 5C and Fig B in S2 Fig). Comparing the metabolic profiles of wild-type and *Mstn* ^−/−^ muscle following starvation revealed the *Mstn* ^−/−^ muscle contained lower amounts of lactate, creatine/phosphocreatine and taurine compared to starved wild-type muscle (Fig 5D and Fig C in S2 Fig). Following starvation, the biochemical signature characteristic of *Mstn* ^−/−^ muscle appeared to normalize towards the wild-type fed muscle profile. Anserine and lactate in the *Mstn* ^−/−^ tissue was partially reduced to more closely resemble concentrations observed in the wild-type muscle (Fig 5E and 5H). Creatine/phosphocreatine remained lower in the starved *Mstn* ^−/−^ muscle while taurine was observed to decrease in comparison to the fed wild-type muscle.

### Serum/glucocorticoid induced kinase 1 (SGK1) a putative regulator of protein synthesis *Mstn* ^*−/−*^ muscle

**Fig 6:**
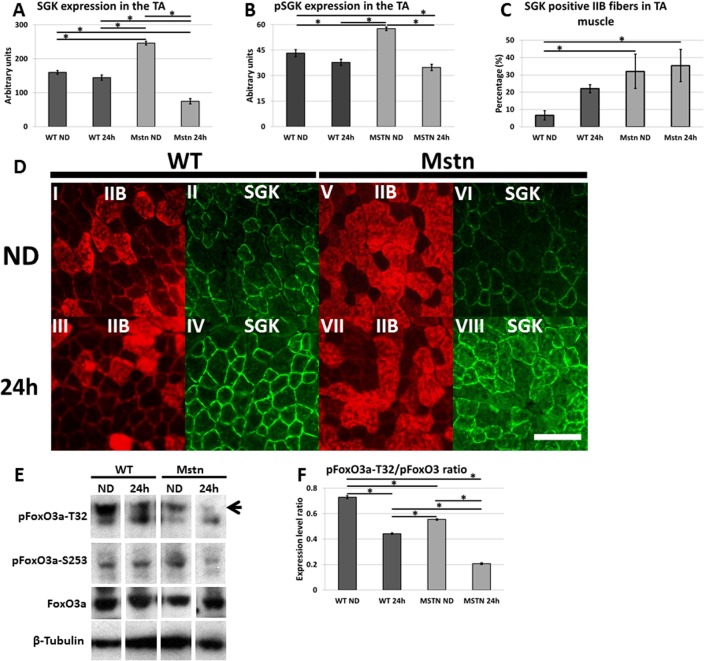
Expression of SGK1 in wild-type and *Mstn* ^*−/−*^ Tibialis Anterior (TA) before and after acute 24 starvation. (A) Western blot and scanning densitometry analysis of 54kD band. (B) (D) Altered profile of SGK1 expression in IIB fibres of *Mstn* ^*−/−*^ TA muscle. (C) Immunochemistry for the expression of MCH IIB and SGK1 (Scale bar = 100μm). (E) Western blot analysis of phosphorylated FoxO3a at residues T32 and S253 from the TA muscles and the influence of starvation. Phosphorylation at T32 indicated by arrow marking the upper band. (F) Quantification of pFoxO3a-T32/ FoxO3a (Two-way ANOVA; *p<0.05).

Our findings that Akt1 activity was insufficient to maintain protein synthesis as well as prevent the activation of the catabolic process in the hypertrophic animals lead to us seeking an alternative pathway responsible for these actions. In a recent elegant study, Andres-Mateos et al. (2013) have described the action of SGK-1, a molecule closely related to Akt1, as a crucial mediator of muscle mass maintenance during mammalian hibernation [23]. We profiled the distribution of SGK1 in muscle from fed and starved animals. Immunohistology showed a SGK1 distribution profile very similar to that for protein synthesis; very few IIB fibres from fed control animals displayed high levels of SGK1 whereas significantly higher proportions expressed the epitope in the same fibres in muscles from *Mstn* ^*−/−*^ animals (Fig 6A–6C). However the proportion of fibres expressing SGK1 did not change significantly after starvation. We also quantified the major 54kD band using Western blotting and densitometry and found higher levels of SGK1 in fed *Mstn* ^*−/−*^ muscle compared to similarly treated wild-type muscle. Significantly we found that starvation induced a significant drop of 70% in the Mstn genotype but only a 9% (non-significant) decrease in wild-types (Fig 6A). An identical profile was discovered while profiling the abundance of active (phosphorylated SGK (Fig 6B and Table 7a-c in S1 Dataset). We extended this study by examining the activation of FoxO3a, a target of SGK1. Here we found that relative to the levels of total FoxO3a, the amount of phosphorylated (inactive) protein through modification at residues T32 or S253 in the TA decreased following starvation more prominently in *Mstn* ^*−/−*^ through starvation than in wild-types (Fig 6E–6F and Table 7d in S1 Dataset).

### Starvation and hypertrophic muscle function

**Fig 7:**
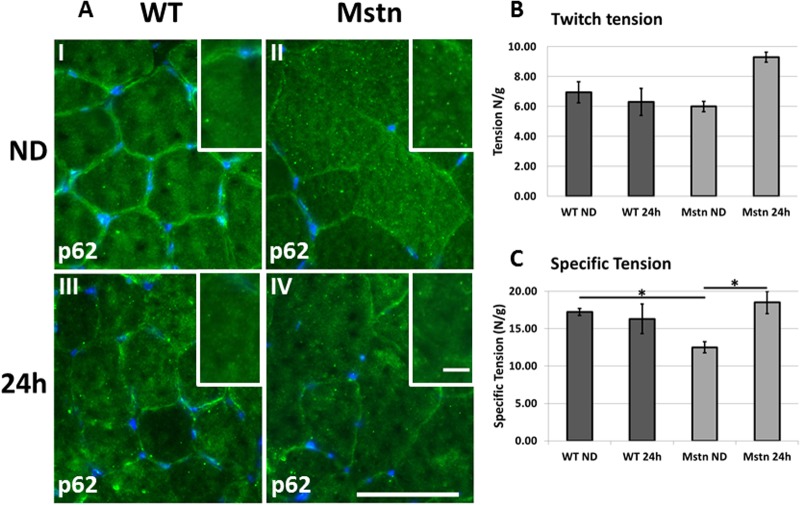
Intra-muscular bodies and muscle function. (A) Immunohistochemical analysis of p62 expression in the EDL of wild-type and *Mstn* ^*−/−*^ mice. (AI) Wild-type mice on normal diet show very few punctate p62 positive bodies in any fibre whereas many appear after starvation (AII). (AIII) Normal diet *Mstn* ^*−/−*^ EDL fibres show large number of punctate p62 bodies predominantly in the large fibres which almost all disappear after starvation (IV) (Scale bar 75μm). Inset images shows high magnification images of p62 staining (scale bar 10μm). (B) Quantification of (B) twitch tension and (C) specific tension of the EDL from wild-type and *Mstn* ^*−/−*^ before and after starvation. (Two-way ANOVA; *p<0.05).

We and others have demonstrated that muscle function in *Mstn* ^*−/−*^ animals is compromised possible to the development of protein aggregates in the contractile filaments that generate tension [14]. Our results here show that autophagic process is activated to a higher degree in *Mstn* ^*−/−*^ muscle compared to wild-types. We determined if there was an accumulation of intracellular material that would necessitate continual autophagy. We therefore examined the formation of microscopic granules containing p62 which mark accumulated aberrant protein material [24]. We found the extensive presence of punctate p62 foci in the enlarged (type IIb) fibres from *Mstn* ^*−/−*^ animals (Fig 7A). Importantly small fibres in the same muscle displayed few if any such foci. Following starvation, there was a significant decrease in the abundance of the punctate p62 particles in *Mstn* ^*−/−*^. Interestingly there was almost the opposing outcome following starvation of normal mice. Here there were very few punctate p62 positive events in the fed mice which increased after 24h of starvation (Fig 7A). Lastly we examined the force generation profile of muscle from both genotypes before and after 24h of starvation. As previously reported we found that the even though the EDL of the Mstn-/- muscle was enlarged, its specific tension profile was significantly lower than that of the wild-type counterpart (Fig 7C). But interestingly starvation had an opposing outcome on the two genotypes. In the wild-types, acute calorie restriction caused a drop in the specific tension whereas it had the contrary outcome in the *Mstn* ^*−/−*^. The level of tension generated by the EDL of the *Mstn* ^*−/−*^ after starvation was no different to the wild-type counterpart raised on a normal diet (Fig 7C). Therefore starvation of the hypertrophic muscled mouse acts to normalise the functional deficit seen at the fed state.

## Discussion

Skeletal muscle mass and composition are influenced by numerous factors including exercise, diet, and age. During the normal adaptive process, muscle undergoes changes while maintaining its functional capability, a concept enshrined in symmorphosis. We suggest that the myostatin null phenotype is a perfect platform to test key elements of symmorphosis since muscle enlargement occurs to levels that are not permitted through adaptive processes. Here we show that muscle enlargement beyond the normal physiological range is accompanied by detrimental changes in function as well as altered programme of development and response to environmental stress (starvation).

We show that acute starvation leads to identical levels of weight loss in both the wild-type and *Mstn* ^*−/−*^ mice; a decrease of over 20% in 24h. These are far higher levels than detected in humans after a similar time interval and are predicted by the inverse relationship between metabolic rate and body size in mammals [25]. However a major difference in the response to starvation between the genotypes was in the identity of the tissue undergoing catabolism; in *Mstn* ^*−/−*^ mice all muscle groups identified had undergone noteworthy weight loss contrasting the non-significant mass decrease in wild-types. This could be explained by the decreased adipose content in *Mstn* ^*−/−*^ mice compared to wild-types [26]. A number of molecules (including glucagon, epinephrine and adrenocorticotropic hormone) have been shown to increase fatty acid mobilisation from adipose tissues to fuel the body during starvation. Under normal physiological conditions, adipose tissue produces molecules (including leptin) that support the anabolic processes in skeletal muscle [27]. Therefore the lack of high levels adipose tissue and the consequential decrease in anabolic signalling in the *Mstn* ^*−/−*^ could explain why muscle undergoes premature myo-catabolism during acute starvation. Nevertheless, muscle from wild-type animals does undergo wasting after a prolonged period [28].

Acute starvation showed a profound difference in the rate of muscle loss between the two genotypes. *Mstn* ^*−/−*^ lost muscle mass in all groups examined earlier and more rapidly than wild-types. There was a strong correlation between the degree of muscle enlargement following myostatin deletion and mass loss due to starvation (Gastrocnemius>Soleus>TA>EDL). It is worth comparing the rate of muscle loss through differing regimes that induce atrophy. The TA in wild-types decreased by 9% per day (whereas the *Mstn* ^*−/−*^ lost twice that amount). Chronic food restriction results in a decrease of only 0.7%/day in TA from wild-types and 1.2% in *Mstn* ^*−/−*^[28]. Denervation is a powerful inducer of TA catabolism (2.3%/day) but is not influenced by myostatin [29]. On the other hand immobilisation leads to a similar rate of muscle loss as chronic food restriction (0.8%/day) but can be completely reversed by interfering with myostatin signalling [29].

Numerous atrophy inducing conditions have shown that fast fibres undergo CSA loss more rapidly than slow fibres [30, 31]. This relationship was sustained during acute starvation in soleus but not the EDL or the TA of wild-type mice but not in any of the muscles from the *Mstn* ^*−/−*^ mice. Therefore the relationship between MHC fibre type and atrophy is guided by muscle identity and genotype. However we did detect a relationship between fibre-type hypertrophy following myostatin deletion and catabolism; those fibres that had undergone the greatest increase displayed the largest decreases in CSA. Therefore at both macro (whole muscle) and cellular level there was a relationship between degree of enlargement and mass decrease in response to starvation. Interestingly there was a relation when fibre atrophy was examined at the metabolic level. In this context we found oxidative fibres were more resistant compared to non-oxidative but only in wild-type mice which would concur with results from a number of laboratories who have shown that oxidative status protects against autophagy [32]. In contrast both groups of muscle fibres from *Mstn* ^*−/−*^ were prone to atrophy which again is compatible with the greater non-oxidative status of muscle in this mutant [14].

Our investigations into starvation induced muscle loss show both qualitative and quantitative difference at the mechanistic level between the two genotypes. The expression of *FoxO1*, a regulator of both proteosome mediated protein degradation and autophagy was not only induced to a greater level but also for an extended period in the *Mstn* ^*−/−*^ compared to wild-types. A similar pattern was found in the expression of *MuRF1* and *Atrogin*, which encode for key E3 class ubiquitin ligases. The up-regulation of *Atrogin* reported in this study is interesting in the context of other wasting models which have reported unchanged expression after chronic starvation [28], as well as limb immobilisation [29] and surprisingly decreased abundance after denervation [29].

Similarly, landscaping the role of autophagy across divergent wasting models reveals differing levels of involvement of this process. We show that at the molecular level increases in the expression of key genes in both genotypes at 12 hours and a further increase thereafter to 24h. Therefore autophagy plays a role on acute starvation mediated muscle loss. Again it is worth contrasting how this differs in other models; no involvement after immobilisation, and a genotype specific involvement (*Mstn* ^*−/−*^ only) after acute starvation [28, 29]. Interestingly we show that basal levels of autophagy at the protein level (lipidation of LC3) were significantly higher in the muscle from *Mstn* ^*−/−*^ animals indicating the presence of unwanted material in the resting condition. This is also reflected in the presence of punctate p62 containing bodies in the resting Type IIB fibres in the muscle of *Mstn* ^*−/−*^ mice, which form in protein aggregates [24]. Importantly these aggregates were completely cleared from *Mstn* ^*−/−*^ following acute starvation whereas they appeared for the first time in the muscle of wild-type animals. It is an attractive proposition to contemplate that the reduced force associated with *Mstn* ^*−/−*^ mice develops as a consequence of aberrant protein aggregates in a manner reminiscent to the many myopathies [33, 34]. Furthermore the restoration of normal muscle function in the *Mstn* ^*−/−*^ muscle through starvation induced autophagy is similar to that in the mouse model for Duchenne and Bethlem/Ullrich muscular dystrophy and further exemplifies the necessity to clear aberrant proteins in order to support muscle contraction [35, 36]. In contrast the wild-type animals showed an increasing trend in punctate p62 containing bodies together with a decrease in specific tension (albeit not reaching significant levels).

At the biochemical level, muscle metabolite variation was observed in the gastrocnemius between the two genotypes with greater anserine and lactate in the *Mstn* ^−/−^ muscle and lower creatine/phosphocreatine compared to their wild-type equivalents. Greater lactate and lower phosphocreatine/creatine abundance in the *Mstn* ^−/−^ muscle reflect differences in the energy capacity of this tissue between the genotypes. Lactate is a product of the anaerobic glycolytic system and is consistent with the known glycolytic muscle phenotype of *Mstn* ^−/−^ animals [12, 37]. Phosphocreatine is an energy store providing short-term replenishment of ATP to support cellular homeostasis. Lower basal phosphocreatine/creatine in the *Mstn* ^−/−^ muscle may contribute to the reduced functionality of this tissue compared to that of wild-type animals. Importantly we show the high ratio of lactate to phosphocreatine/creatine in the muscle of the *Mstn* ^−/−^ animals, which is conducive for channelling of carbon into anabolic programmes rather than for the generation of energy to support tissue function. This would be compatible with the phenotype of the *Mstn* ^−/−^ mice that use carbon to generate skeletal muscle mass. We invite parallels to be drawn between the metabolic profile displayed by the *Mstn* ^−/−^ muscle and that often found in cancer known as the ‘Warburg Effect’ in which cells adopt metabolic strategies that rely on glycolysis to ensure biomass production even in aerobic conditions [38]. As expected the *Mstn* ^−/−^ animals respond to starvation by withdrawing from metabolic programmes that support anabolism by decreasing lactate content with concomitant decreases in anserine. These starvation-associated changes result in a biomolecular phenotype that more closely resembles that of WT muscle. This is consistent with the restoration of muscle function in terms of force following starvation in these animals.

*Mstn* ^−/−^ muscle contains greater amounts of anserine, an aminoacyl-imadazole dipeptide. Anserine is formed in skeletal muscle, it activates myosin ATPases [39] and can regulate muscle phosphorylase activity [40]. Anserine has a H^+^ buffering capacity [41, 42] and the higher concentration may have a role in regulating lower intracellular pH, as a result of lactate production, in glycolytic *Mstn* ^−/−^ muscle. Our results are in accordance with previous studies showing that anserine is found is higher concentrations in glycolytic muscles [43], [44]. Anserine also possesses antioxidant activity and greater amounts in the *Mstn* ^−/−^ muscle may reflect lower levels of oxidative stress [45]. In agreement, *Mstn* ^−/−^ muscle contain a lower abundance of oxidative fibres and therefore has potential to generate fewer reactive oxygen species. This may imply that highly glycolytic muscles are more stable than oxidative ones.

From the metabolic profiles, the wild-type mice appear better adapted than the *Mstn* ^−/−^ mice to buffer the metabolic consequences of starvation and exhibit a markedly varied response to this challenge. The enhanced metabolic flexibility of the wild-type mice most likely explains this observation. The animals with altered muscle metabolism following starvation had increased lactate, phosphocreatine/creatine and taurine. These represent three strategies for maintaining energy levels with increased consumption of glycogen (lactate), phosphocreatine, and protein (taurine). It has been previously reported that taurine content increases with the oxidative capacity of muscle [46] which is in accordance with the oxidative phenotype of wild-type starved mice breaking down muscle protein for energy.

One of the most intriguing aspects of our data is that related to the canonical Akt pathway and its role during acute starvation. We show that in wild-type mice, starvation leads to a complete loss of activated Akt and commensurate with an increase in the autophagic and proteosome programme [47]. However from studying *Mstn* ^*−/−*^ muscle two surprising discoveries were made: Firstly that basal levels of active Akt were lower in the hypertrophic animal than in wild-types either at S473 or T308 (Fig 3) and secondly that starvation had no effect on this protein even through activity of its targets (S6 and 4EBP1) as well as the rate of protein synthesis as judged by using the SUnSET protocol were decreased. We suggest that Akt plays only a minor role in hypertrophic muscle development. Indeed our finding resonant with studies investigating muscle growth induced by IGF-1 which also results in muscle hypertrophy but no change in Akt activation [48]. The lower levels of resting Akt in *Mstn* ^*−/−*^ is surprising since it has been demonstrated that Smad2/3, downstream components in myostain signalling, directly interacts and inhibits the activity of Akt [49]. We suggest these differences reconciled by taking into account that our studies were performed in adult muscle fibres whereas the work of Trendelenberg was performed on myoblasts [49]. Nevertheless we show here that Akt does not influence autophagy, proteosome regulators or protein synthesis in adult muscle in the absence of myostatin when challenged by starvation.

We suggest that SGK-1 a molecule related to Akt not only in terms of structure but also specificity is an important regulator of hypertrophic muscle development in the absence of myostatin. Elegant work from the Cohn group have provided a wealth of data backing a central role for SGK-1 rather than Akt in muscle homeostasis and atrophy through its ability to promote protein synthesis and inhibiting the catabolic activity of FoxO proteins [23, 29]. Our work furthers the notion that SGK-1 plays a significant role in muscle hypertrophy. We show that levels and activity of this protein are elevated in the muscle of *Mstn* ^*−/−*^ mice. Moreover, there is an increase in the number of hypertrophic fibres that express the protein. Lastly, SGK-1 expression drops as does its activity (identified through increased levels of un-modified FoxO3a) significantly after starvation, providing additional evidence for the involvement of this protein during starvation.

## Conclusions

In summary our work shows that the hypertrophic muscle that develops following the genetic ablation of myostatin is prone to catabolism. We suggest that it has a number of mechanisms primed for this outcome including active autophagy. However the consequence of catabolism in the *Mstn* ^*−/−*^ is beneficial in that muscle function is restored. We suggest that starvation reinstates the function of muscle back to its normal symmorphosis range. We provide evidence that this takes places through a coordinated combination among decreased protein synthesis, augmented autophagy and accelerated proteolysis in hypertrophic muscles from *Mstn* ^−/−^ mice. In contrast starvation is not beneficial to wild-type animals. Importantly our work shows a major role for SGK1 in muscle hypertrophy therefore identifies it rather than Akt as a regulator of muscle mass. Therefore the future development of SGK1 mimetics could represent a novel class of molecules used to prevent muscle wasting in clinical setting such as cancer and after appetite loss as well as to attenuate the effects of sacropenia.

## Supporting Information

S1 Dataset Primer sequence, quantitative measures and statistical evaluations. (DOCX)

S1 Fig Quantification of p62, 4ebp6 and S6 protein levels. (TIF)

S2 Fig Pair-wise comparisons of the metabolic profiles obtained from gastrocnemius muscle from wild-type and *Mstn* ^−/−^ mice under fed and starved states. Colour loadings plots shown for (A) PC1 of the model comparing wild-type fed vs wild-type starved; (B) PC1 of the model comparing *Mstn* ^−/−^ fed vs *Mstn* ^−/−^ starved and (C) PC1 of the model comparing wild-type starved vs *Mstn* ^−/−^ starved. Product of PC loadings with standard deviation of the entire data set, coloured by the square of the PC loading.
(TIF)
