# Correction: Symmorphosis through Dietary Regulation: A Combinatorial Role for Proteolysis, Autophagy and Protein Synthesis in Normalising Muscle Metabolism and Function of Hypertrophic Mice after Acute Starvation

**DOI:** 10.1371/journal.pone.0128731

**Published:** 2015-05-14

**Authors:** Henry Collins-Hooper, Roberta Sartori, Natasa Giallourou, Antonios Matsakas, Robert Mitchell, Helen P. Makarenkova, Hannah Flasskamp, Raymond Macharia, Steve Ray, Jonathan R. Swann, Marco Sandri, Ketan Patel

The sixth author’s name is spelled incorrectly. The correct name is: Helen P. Makarenkova. The correct citation is: Collins-Hooper H, Sartori R, Giallourou N, Matsakas A, Mitchell R, Makarenkova HP, et al. (2015) Symmorphosis through Dietary Regulation: A Combinatorial Role for Proteolysis, Autophagy and Protein Synthesis in Normalising Muscle Metabolism and Function of Hypertrophic Mice after Acute Starvation. PLoS ONE 10(3): e0120524. doi:10.1371/journal.pone.0120524

## References

[1] Collins-Hooper H, Sartori R, Giallourou N, Matsakas A, Mitchell R, Makarenkova H, et al (2015) Symmorphosis through Dietary Regulation: A Combinatorial Role for Proteolysis, Autophagy and Protein Synthesis in Normalising Muscle Metabolism and Function of Hypertrophic Mice after Acute Starvation. PLoS ONE 10(3): e0120524 doi: 10.1371/journal.pone.0120524 25807490 10.1371/journal.pone.0120524 PMC4373938

