# Supplementary material for: Symmorphosis through Dietary Regulation: A Combinatorial Role for Proteolysis, Autophagy and Protein Synthesis in Normalising Muscle Metabolism and Function of Hypertrophic Mice after Acute Starvation
Source: PLoS One. 2015 Mar 25;10(3):e0120524. doi: 10.1371/journal.pone.0120524 (PMC4373938; doi:10.1371/journal.pone.0120524)
Supplement: S1 Dataset — (DOCX) [file pone.0120524.s001.docx]

# Supporting Data

**Table 1 qPCR primer sequence (all mouse)**

|  | **Forward Primer** | **Reverse Primer** |
| --- | --- | --- |
| **4EBP1** | CCTCCTTGTGCCTCTGTCTA | GCCTAAGGAAAGATGGGTGT |
| **FoxO1** | GCTGGGTGTCAGGCTAAGAG | AGGGGTGAAGGGCATCT |
| **MuRF1** | ACCTGCTGGTGGAAAACATC | CTTCGTGTTCCTTGCACATC |
| **Atrogin-1** | GCAAACACTGCCACATTCTCTC | CTTGAGGGGAAAGTGAGACG |
| **Bnip3** | TTCCACTAGCACCTTCTGATGA | GAACACCGCATTTACAGAACAA |
| **Beclin1** | TGAATGAGGATGACAGTGAGCA | CACCTGGTTCTCCACACTCTTG |
| **LC3b** | CACTGCTCTGTCTTGTGTAGGTTG | TCGTTGTGCCTTTATTAGTGCATC |
| **ATF4** | TCCTGAACAGCGAAGTGTTG | ACCCATGAGGTTTCAAGTGC |
| **CathepsinL** | GTGGACTGTTCTCACGCTCAAG | TCCGTCCTTCGCTTCATAGG |
| **Vps34** | TGTCAGATGAGGAGGCTGTG | CCAGGCACGACGTAACTTCT |
| **P62** | CCCAGTGTCTTGGCATTCTT | AGGGAAAGCAGAGGAAGCTC |
| **Gadd34** | AGAGAAGACCAAGGGACGTG | CAGCAAGGAATGGACTGTG |
| **Mul1** | AGGGCATTCTTTCAGAAGCA | GGGGTGGAACTTCTCGTACA |
| **GAPDH** | CACCATCTTCCAGGAGCGAG | CCTTCTCCATGGTGGTGAAGAC |

# Table 2a; Animal weights over the 24h starvation period

|  | **WT ND (g)** | **WT 12h (g)** | **WT 24h (g)** | **Mstn ND (g)** | **Mstn 12h (g)** | **Mstn 24h (g)** |
| --- | --- | --- | --- | --- | --- | --- |
|  | 21.023 | 18.553 | 16.430 | 26.336 | 23.423 | 20.438 |
| **Anova** |  |  |  |  |  |  |
| **WT ND** |  | ns | * | * | ns | ns |
| **WT 12h** |  |  | ns | * | * | ns |
| **WT 24h** |  |  |  | * | * | * |
| **Mstn ND** |  |  |  |  | * | * |
| **Mstn 12h** |  |  |  |  |  | * |

# Table 2b; Individual muscle weights

|  | **EDL (g)** | **Soleus (g)** | **TA (g)** | **Gastrocnemius (g)** |
| --- | --- | --- | --- | --- |
| **WT ND** | 0.0098 | 0.0068 | 0.0372 | 0.1015 |
| **WT 12h** | 0.0100 | 0.0067 | 0.0350 | 0.1013 |
| **WT 24h** | 0.0094 | 0.0069 | 0.0338 | 0.0929 |
| **Mstn ND** | 0.0155 | 0.0119 | 0.0632 | 0.1838 |
| **Mstn 12h** | 0.0135 | 0.0097 | 0.0551 | 0.1821 |
| **Mstn 24h** | 0.0130 | 0.0095 | 0.0520 | 0.1459 |
| **Anova** |  |  |  |  |
| **WT ND vs WT 12h** | ns | ns | ns | ns |
| **WT ND vs WT 24h** | ns | ns | ns | ns |
| **WT ND vs Mstn ND** | * | * | * | * |
| **WT ND vs Mstn 12h** | * | * | * | * |
| **WT ND vs Mstn 24h** | * | * | * | * |
| **WT 12h vs WT 24h** | ns | ns | ns | ns |
| **WT 12h vs Mstn ND** | * | * | * | * |
| **WT 12h vs Mstn 12h** | * | * | * | * |
| **WT 12h vs Mstn 24h** | * | * | * | * |
| **WT 24h vs Mstn ND** | * | * | * | * |
| **WT 24h vs Mstn 12h** | * | * | * | * |
| **WT 24h vs Mstn 24h** | * | * | * | * |
| **Mstn ND vs Mstn 12h** | ns | * | * | ns |
| **Mstn ND vs Mstn 24h** | * | * | * | * |
| **Mstn 12h vs Mstn 24h** | ns | ns | ns | * |

# Table 3a; Extensor digitorum longus (EDL) cross-sectional area measurements

|  | **IIA (µm^2^)** | **IIX (µm^2^)** | **IIB (µm^2^)** |
| --- | --- | --- | --- |
| **WT ND** | 442.95 | 629.26 | 1557.7 |
| **WT 24h** | 398.06 | 496.39 | 1377.4 |
| **Mstn ND** | 657.02 | 995.33 | 1999.0 |
| **Mstn 24h** | 507.19 | 604.26 | 1466.3 |
| **Anova** |  |  |  |
| **WT ND vs WT 24h** | * | * | * |
| **WT ND vs Mstn ND** | * | * | * |
| **WT ND vs Mstn 24h** | * | ns | ns |
| **WT 24h vs Mstn ND** | * | ns | * |
| **WT 24h vs Mstn 24h** | * | * | ns |
| **Mstn ND vs Mstn 24h** | * | * | * |

# Table 3b; Soleus cross-sectional area measurements

|  | **I (µm^2^)** | **IIA (µm^2^)** | **IIX (µm^2^)** | **IIB (µm^2^)** |
| --- | --- | --- | --- | --- |
| **WT ND** | 1767.7 | 1275.7 | 1586.3 | 0.0 |
| **WT 24h** | 1790.9 | 1218.6 | 1272.7 | 0.0 |
| **Mstn ND** | 2732.8 | 2031.3 | 1419.0 | 2712.0 |
| **Mstn 24h** | 2158.4 | 1457.4 | 1449.4 | 1862.7 |
| **Anova** |  |  |  |  |
| **WT ND vs WT 24h** | ns | ns | * |  |
| **WT ND vs Mstn ND** | * | * | ns |  |
| **WT ND vs Mstn 24h** | * | * | ns |  |
| **WT 24h vs Mstn ND** | * | * | ns |  |
| **WT 24h vs Mstn 24h** | * | * | ns |  |
| **Mstn ND vs Mstn 24h** | * | * | ns | * |

**Table 3c; Tibialis anterior cross-sectional area measurements**

|  | **IIA (µm^2^)** | **IIX (µm^2^)** | **IIB (µm^2^)** |
| --- | --- | --- | --- |
| **WT ND** | 945.70 | 1536.8 | 2523.7 |
| **WT 24h** | 814.25 | 993.85 | 2321.2 |
| **Mstn ND** | 1216.0 | 1284.6 | 2523.1 |
| **Mstn 24h** | 1055.4 | 1220.3 | 2301.2 |
| **Anova** |  |  |  |
| **WT ND vs WT 24h** | * | * | ns |
| **WT ND vs Mstn ND** | * | * | ns |
| **WT ND vs Mstn 24h** | * | * | ns |
| **WT 24h vs Mstn ND** | * | * | ns |
| **WT 24h vs Mstn 24h** | * | * | ns |
| **Mstn ND vs Mstn 24h** | * | ns | ns |

# Table 3d; Percentage of oxidative fibres in the EDL

|  | **WT ND** | **WT 24h** | **Mstn ND** | **Mstn 24h** |
| --- | --- | --- | --- | --- |
|  | 46.135 | 31.853 | 36.306 | 23.418 |
| **Anova** |  |  |  |  |
| **WT ND** |  | * | * | * |
| **WT 24h** |  |  | ns | ns |
| **Mstn ND** |  |  |  | * |

# Table 4a; RT-PCR on the tibialis anterior (TA)

|  | **MuRF1** | **Atrogin** | **4EBP1** | **Gadd34** | **LC3b** | **p62** | **Bnip3** | **Beclin** | **CathepsinL** | **ATF4** | **Vps34** |
| --- | --- | --- | --- | --- | --- | --- | --- | --- | --- | --- | --- |
| **WT ND** | 0.9633 | 0.9835 | 1.0566 | 1.1085 | 0.6668 | 1.0929 | 0.9919 | 1.0329 | 1.0676 | 1.0665 | 0.9226 |
| **WT 12h** | 16.836 | 8.0738 | 7.5820 | 5.7753 | 2.8473 | 2.8715 | 2.4928 | 1.2840 | 1.8945 | 2.6538 | 1.1735 |
| **WT 24h** | 17.765 | 12.55325 | 7.2845 | 7.4448 | 4.1775 | 4.4685 | 4.2863 | 1.0103 | 2.8520 | 2.2003 | 1.6538 |
| **Mstn ND** | 0.9710 | 0.9643 | 1.1406 | 1.0141 | 1.5134 | 1.1331 | 0.7990 | 0.7592 | 0.9757 | 1.0189 | 0.8774 |
| **Mstn 12h** | 21.179 | 9.5538 | 8.8815 | 10.723 | 3.2108 | 2.9403 | 2.4185 | 1.0825 | 1.9058 | 2.3645 | 1.5440 |
| **Mstn 24h** | 36.052 | 17.331 | 15.201 | 15.393 | 5.9335 | 6.2308 | 5.6230 | 1.0850 | 2.7833 | 3.2965 | 2.1363 |
| **Anova** |  |  |  |  |  |  |  |  |  |  |  |
| **WT ND vs WT 12h** | * | * | * | * | * | * | * | ns | * | * | ns |
| **WT ND vs WT 24h** | * | * | * | * | * | * | * | ns | * | * | * |
| **WT ND vs Mstn ND** | ns | ns | ns | ns | ns | ns | ns | ns | ns | ns | ns |
| **WT ND vs Mstn 12h** | * | * | * | * | * | * | ns | ns | * | * | * |
| **WT ND vs Mstn 24h** | * | * | * | * | * | * | * | ns | * | * | * |
| **WT 12h vs WT 24h** | ns | ns | ns | ns | ns | * | * | ns | * | ns | * |
| **WT 12h vs Mstn ND** | * | * | * | * | ns | * | * | * | * | * | ns |
| **WT 12h vs Mstn 12h** | ns | ns | ns | * | ns | ns | ns | ns | ns | ns | ns |
| **WT 12h vs Mstn 24h** | * | * | * | * | * | * | * | ns | * | * | * |
| **WT 24h vs Mstn ND** | * | * | * | * | * | * | * | ns | * | * | * |
| **WT 24h vs Mstn 12h** | ns | ns | ns | ns | ns | * | * | ns | * | ns | ns |
| **WT 24h vs Mstn 24h** | * | ns | * | * | * | * | ns | ns | ns | * | * |
| **Mstn ND vs Mstn 12h** | * | * | * | * | * | * | * | ns | * | * | * |
| **Mstn ND vs Mstn 24h** | * | * | * | * | * | * | * | ns | * | * | * |
| **Mstn 12h vs Mstn 24h** | * | * | * | * | * | * | * | ns | ns | * | * |

# Table 4b; LC3 I/II and pAkt-s473/Akt expression ratios

|  | **LC3 I/II** | **pAkt/Akt** |
| --- | --- | --- |
| **WT ND** | 0.0510 | 0.6900 |
| **WT 12h** | 1.3600 | 0.3067 |
| **WT 24h** | 3.2267 | 0.13133333 |
| **Mstn ND** | 0.8275 | 0.3967 |
| **Mstn 12h** | 1.8400 | 0.3667 |
| **Mstn 24h** | 1.1500 | 0.5077 |
| **Anova** |  |  |
| **WT ND vs WT 12h** | * | * |
| **WT ND vs WT 24h** | * | * |
| **WT ND vs Mstn ND** | * | * |
| **WT ND vs Mstn 12h** | * | * |
| **WT ND vs Mstn 24h** | * | * |
| **WT 12h vs WT 24h** | * | * |
| **WT 12h vs Mstn ND** | ns | ns |
| **WT 12h vs Mstn 12h** | ns | ns |
| **WT 12h vs Mstn 24h** | ns | * |
| **WT 24h vs Mstn ND** | * | * |
| **WT 24h vs Mstn 12h** | * | * |
| **WT 24h vs Mstn 24h** | * | * |
| **Mstn ND vs Mstn 12h** | * | ns |
| **Mstn ND vs Mstn 24h** | ns | ns |
| **Mstn 12h vs Mstn 24h** | ns | ns |

# Table 4c; Mul1 RT-PCR on the tibialis anterior (TA)

|  | **WT ND** | **WT 12h** | **WT 24h** | **Mstn ND** | **Mstn 12h** | **Mstn 24h** |
| --- | --- | --- | --- | --- | --- | --- |
| **Mul1** | 0.8494 | 1.2368 | 1.7933 | 0.6614 | 1.2833 | 0.9964 |
| **Anova** |  |  |  |  |  |  |
| **WT ND** |  | ns | * | ns | ns | ns |
| **WT 12h** |  |  | ns | ns | ns | ns |
| **WT 24h** |  |  |  | * | ns | * |
| **Mstn ND** |  |  |  |  | ns | ns |
| **Mstn 12h** |  |  |  |  |  | ns |

Table 5a; FoxO1 RT-PCR on the tibialis anterior (TA)

|  | **WT ND** | **WT 12h** | **WT 24h** | **Mstn ND** | **Mstn 12h** | **Mstn 24h** |
| --- | --- | --- | --- | --- | --- | --- |
| FoxO1 | 0.8718 | 2.7243 | 2.4295 | 0.5301 | 3.6938 | 5.2053 |
| **Anova** |  |  |  |  |  |  |
| **WT ND** |  | * | * | ns | * | * |
| **WT 12h** |  |  | ns | * | * | * |
| **WT 24h** |  |  |  | * | * | * |
| **Mstn ND** |  |  |  |  | * | * |
| **Mstn 12h** |  |  |  |  |  | * |

**Table 5b; p62 protein expression relative to tubulin**

|  | **WT ND** | **WT 24h** | **MSTN ND** | **MSTN 24h** |
| --- | --- | --- | --- | --- |
| **p62** | 1.25 | 3.775 | 3.6 | 8 |
| **Anova** |  |  |  |  |
| **WT ND** |  | * | * | * |
| **WT24H** |  |  | ns | * |
| **Mstn ND** |  |  |  | * |

# Table 5c; p4ebp6/4ebp6 expression ratio (protein)

|  | **WT ND** | **WT 24h** | **MSTN ND** | **MSTN 24h** |
| --- | --- | --- | --- | --- |
| **p4ebp6/4ebp6 ratio** | 0.802 | 0.053333 | 0.4466667 | 0.045 |
| **Anova** |  |  |  |  |
| **WT ND** |  | * | * | * |
| **WT24H** |  |  | * | ns |
| **Mstn ND** |  |  |  | * |

# Table 5d; pS6/S6 expression ratio (protein)

|  | **WT ND** | **WT 24h** | **MSTN ND** | **MSTN 24h** |
| --- | --- | --- | --- | --- |
| **pS6/S6 ratio** | 0.945 | 0 | 0.635 | 0 |
| **Anova** |  |  |  |  |
| **WT ND** |  | * | * | * |
| **WT24H** |  |  | * | ns |
| **Mstn ND** |  |  |  | * |

# Table 6a; Relative puromycin levels in the TA and the gastrocnemius

|  | **TA** | **Gastrocnemius** |
| --- | --- | --- |
| **WT ND** | 68.333 | 160.67 |
| **WT 24h** | 58.333 | 129.00 |
| **MSTN ND** | 216.67 | 224.67 |
| **MSTN 24h** | 120.67 | 152.33 |
| **Anova** |  |  |
| **WT ND vs WT 24h** | ns | ns |
| **WT ND vs Mstn ND** | * | * |
| **WT ND vs Mstn 24h** | * | ns |
| **WT 24h vs Mstn ND** | * | * |
| **WT 24h vs Mstn 24h** | * | ns |
| **Mstn ND vs Mstn 24h** | * | * |

# Table 7a; SGK expression in the TA

|  | **WT ND** | **WT 24h** | **Mstn ND** | **Mstn 24h** |
| --- | --- | --- | --- | --- |
| **SGK** | 159.75 | 144.25 | 245.75 | 74.500 |
| **Anova** |  |  |  |  |
| **WT ND** |  | ns | * | * |
| **WT 24h** |  |  | * | * |
| **Mstn ND** |  |  |  | * |

# Table 7b; pSGK expression in the TA

|  | **WT ND** | **WT 24h** | **MSTN ND** | **MSTN 24h** |
| --- | --- | --- | --- | --- |
| **pSGK** | 43.2 | 37.8 | 57.6 | 34.8 |
| **Anova** |  |  |  |  |
| **WT ND** |  | ns | * | * |
| **WT24H** |  |  | * | ns |
| **Mstn ND** |  |  |  | * |

# Table 7c; Percentage puromycin and SGK positive IIB fibres in the TA

|  | **Puromycin (%)** | **SGK (%)** |
| --- | --- | --- |
| **WT ND** | 16.667 | 6.6667 |
| **WT 24h** | 34.667 | 22.000 |
| **Mstn ND** | 66.000 | 32.000 |
| **Mstn 24h** | 62.667 | 35.333 |
| **Anova** |  |  |
| **WT ND vs WT 24h** | ns | ns |
| **WT ND vs Mstn ND** | * | * |
| **WT ND vs Mstn 24h** | * | * |
| **WT 24h vs Mstn ND** | ns | ns |
| **WT 24h vs Mstn 24h** | ns | ns |
| **Mstn ND vs Mstn 24h** | ns | ns |

**Table 7d; pFoxO3a-T32/pFoxO3 ratio**

|  | **WT ND** | **WT 24h** | **MSTN ND** | **MSTN 24h** |
| --- | --- | --- | --- | --- |
| **pFoxo3a-T32/pFoxO3 ratio** | 0.72967 | 0.442667 | 0.554 | 0.20733333 |
| **Anova** |  |  |  |  |
| **WT ND** |  | * | * | * |
| **WT24H** |  |  | * | * |
| **Mstn ND** |  |  |  | * |
