# Supplementary figures and images for: Symmorphosis through Dietary Regulation: A Combinatorial Role for Proteolysis, Autophagy and Protein Synthesis in Normalising Muscle Metabolism and Function of Hypertrophic Mice after Acute Starvation
Source: PLoS One. 2015 Mar 25;10(3):e0120524. doi: 10.1371/journal.pone.0120524 (PMC4373938; doi:10.1371/journal.pone.0120524)

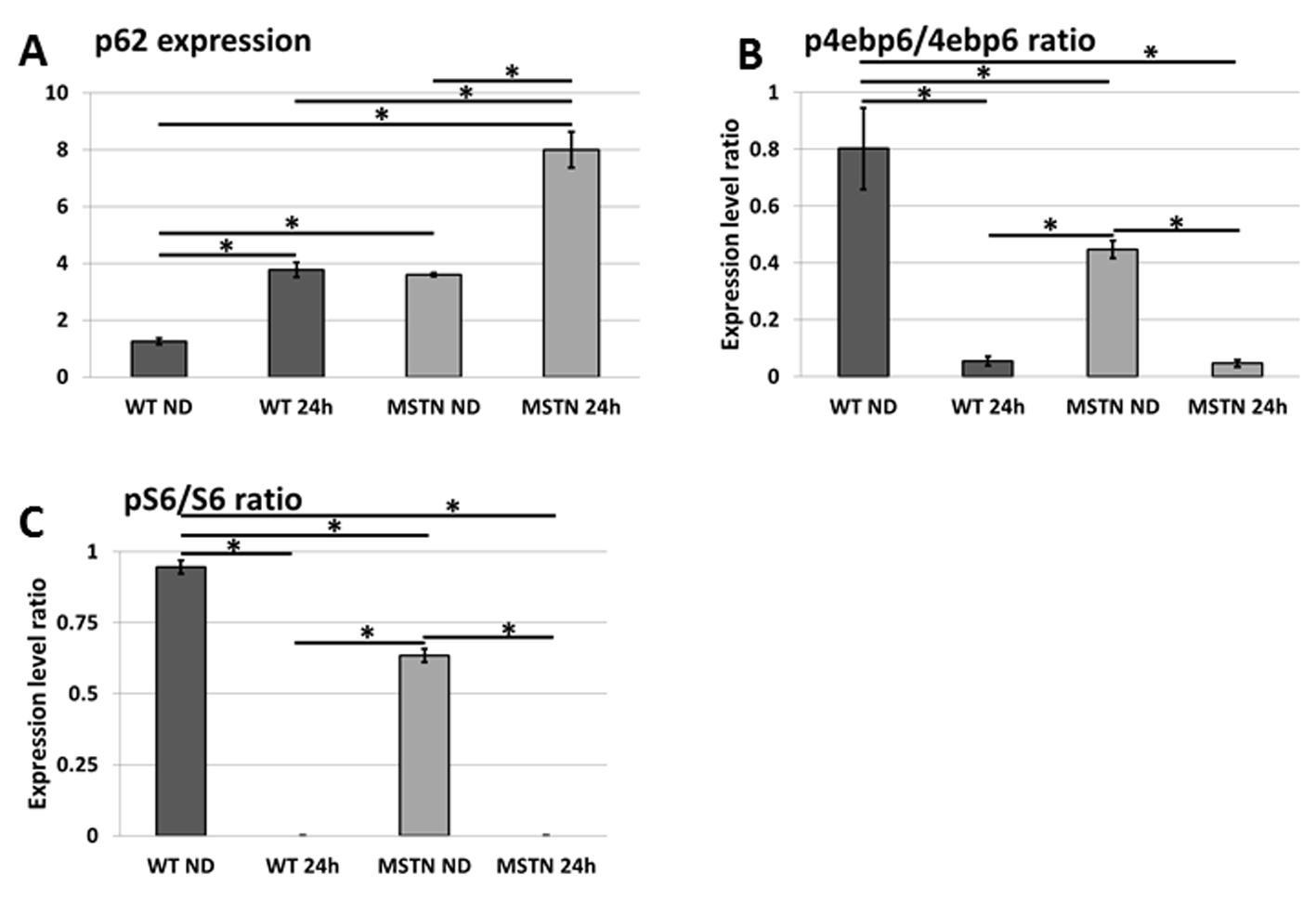

Supplement: S1 Fig — (TIF) [file pone.0120524.s002.tif]

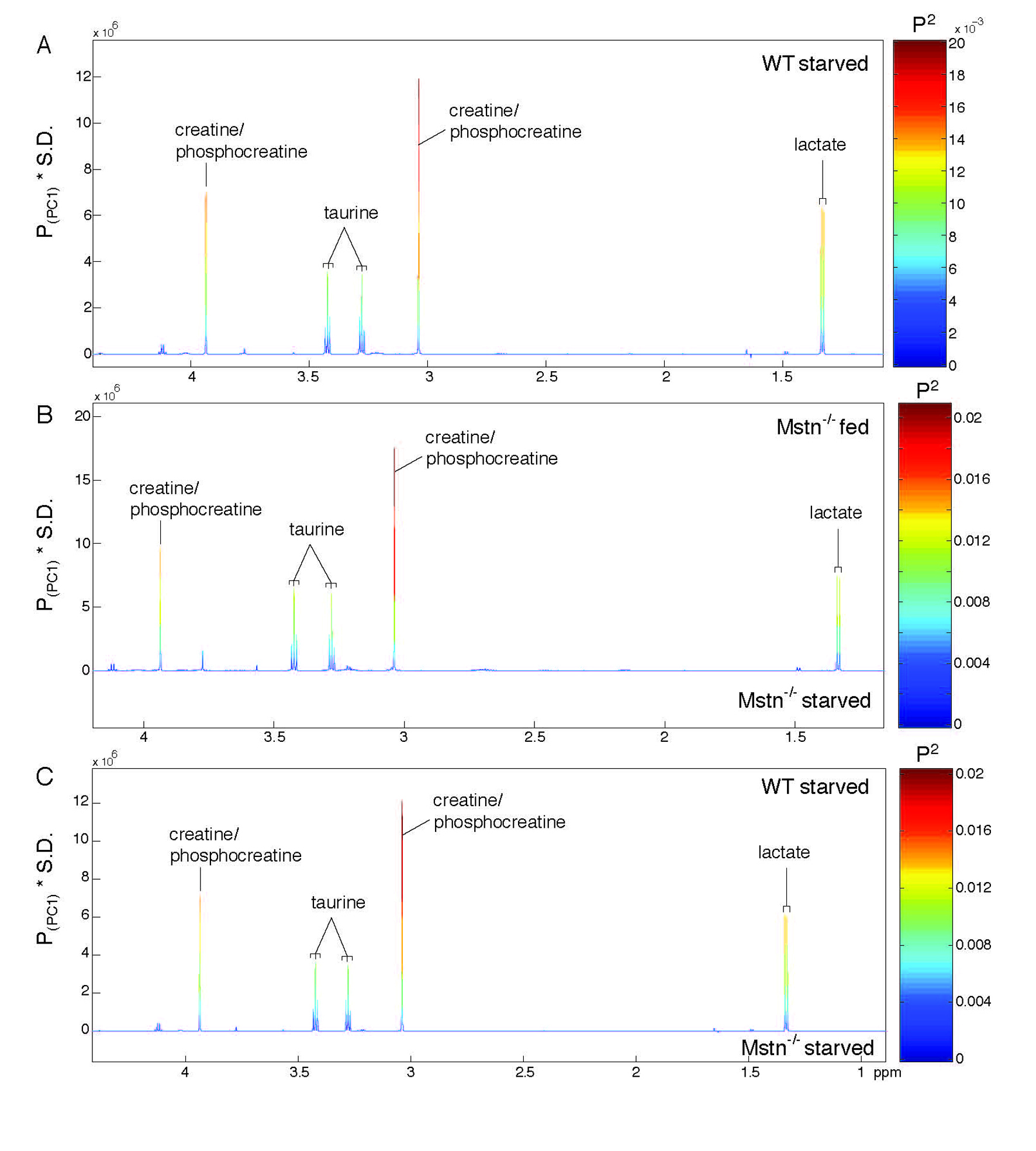

Supplement: S2 Fig — Colour loadings plots shown for (A) PC1 of the model comparing wild-type fed vs wild-type starved; (B) PC1 of the model comparing Mstn −/− fed vs Mstn −/− starved and (C) PC1 of the model comparing wild-type starved vs Mstn −/− starved. Product of PC loadings with standard deviation of the entire data set, coloured by the square of the PC loading. (TIF) [file pone.0120524.s003.tif]
